# Neutrophil/lymphocyte ratio and lymphocyte/monocyte ratio in ulcerative colitis as non-invasive biomarkers of disease activity and severity

**DOI:** 10.1186/s13317-019-0114-8

**Published:** 2019-05-15

**Authors:** Ashraf M. Okba, Mariam M. Amin, Ahmed S. Abdelmoaty, Hend E. Ebada, Amgad H. kamel, Ahmed S. Allam, Omar M. Sobhy

**Affiliations:** 1 Department of Internal Medicine, Clinical Immunology and Allergy, Ain Shams University, Cairo, Egypt; 2 Department of Tropical Medicine, Ain Shams University, Cairo, Egypt; 3 Department of Internal Medicine, Ain Shams University, Cairo, Egypt

**Keywords:** Disease activity, Monocyte–lymphocyte ratio, Neutrophil–lymphocyte ratio, Ulcerative colitis

## Abstract

**Background:**

Apart from endoscopic interventions, readily attainable cost-effective biomarkers for ulcerative colitis (UC) assessment are required. For this purpose, we evaluated differential leucocytic ratio, mainly neutrophil–lymphocyte ratio (NLR) and lymphocyte-monocyte ratio (LMR) as simple available indicators of disease activity in patients with ulcerative colitis.

**Methods:**

Study conducted on 80 UC patients who were classified into two groups of 40 each according to Mayo score and colonoscopic findings. Group 1 (active UC) and group 2 (inactive UC). Another 40 group-matched healthy participants were enrolled. White blood cell count, NLR, LMR, C-reactive protein, and Erythrocyte sedimentation rate were measured and recorded.

**Results:**

Significant elevation of NLR was observed in active UC group compared to inactive UC and controls (2.63 ± 0.43, 1.64 ± 0.25, 1.44 ± 0.19 respectively; p < 0.0001). The optimal NLR cut-off value for active UC was > 1.91, with a sensitivity and a specificity of 90% and 90% respectively. The mean LMRs of active UC was significantly lower compared with inactive UC patients and controls (2.25 ± 0.51, 3.58 ± 0.76, 3.64 ± 0.49 respectively; p < 0.0001). The cut-off value of LMR for determining the disease activity was ≤ 2.88 with a sensitivity of 90% and a specificity of 90%. NLR, LMR, and CRP were found to be significant independent markers for discriminating disease activity (p = 0.000). Besides, NLR was significantly higher in patients with pancolitis and positively correlated with endoscopically severe disease.

**Conclusion:**

NLRs and LMRs are simple non-invasive affordable independent markers of disease activity in UC.

## Introduction

Ulcerative colitis (UC) is a chronic relapsing form of inflammatory bowel disease (IBD) characterized by continuous mucosal inflammation in the innermost layers of the colon and rectum. A primary concern of UC is assessment of intestinal inflammation and evaluation of healing with long-term prognosis [1]. However, it is a difficult challenge and a major area of interest. Despite success in practice, endoscopic and histopathological examination are invasive, costly and have some complications in use [2].

Both mucosal and clinical evaluation are independently essential in ulcerative colitis. Endoscopy may show active mucosal inflammation in absence of clinical manifestations and in the same manner clinical remission is not linked to mucosal cure. Of particular concern is that multiple endoscopic biopsies during clinical remission may increase the risk of UC activity recurrence in the long term. Away from endoscopy, disease severity can be assessed using laboratory investigations and non-invasive radiology [3]. However, previous studies offer contradictory findings about effective noninvasive markers for the detection of mucosal activity in UC [4, 5].

UC activity have been assessed in different studies using laboratory markers as erythrocyte sedimentation rate (ESR) and C-reactive protein (CRP), with sensitivities and specificities ranging between 50 and 60% [6]. Other markers as fecal calprotectin and lactoferrin are more specific and sensitive. However, both of them are costly and not commonly utilized in clinical practice [6, 7]. This indicates a need for simply reachable, efficacious and cost-effective biomarkers for proper management of UC.

Previous research has established the existence of a correlation of both neutrophils and blood mononuclear cells with disease activity. Besides, they can predict disease severity in some diseases such as rheumatoid arthritis and acute coronary syndrome [8, 9]. Undoubtedly, macrophages and neutrophils are interestingly involved in IBD pathophysiology [10]. And during routine clinical visits changes in leukocyte numbers, particularly monocytes, could also be detected early in IBD as biomarkers of inflammation [11, 12].

Extensive research has considered neutrophil–lymphocyte ratio (NLR) as a useful biomarker of systemic inflammation [13, 14] and prediction of mortality in some diseases as malignancy, including gastrointestinal malignancies [15, 16]. According to foregoing studies, the NLR can be considered a new marker for assessment of UC endoscopic activity and severity [17, 18].

Besides, in 2015 Cherfane et al. [19] observed that elevated absolute monocytic count and low lymphocyte to monocyte ratio (LMR) can predict disease activity in UC patients. Hence, this study aimed to clarify the role of differential leucocytic ratio, NLR, and LMR as a simple, cost-effective and available predictors of disease activity in patients with ulcerative colitis.

## Methodology

### Patient selection

This case–control study included eighty patients newly diagnosed as ulcerative colitis. Participants were recruited from the gastroenterology and internal medicine department, Ain Shams University hospital over a period of 2 years (January 2015 to January 2017). We further classified the eighty patients into two groups of forty each based on clinical, laboratory, radiological, endoscopic, and histological findings. Group 1 (40 patients with active disease) and group 2 (40 patients with inactive disease). None of the participants was on medical treatment (e.g. corticosteroids, azathioprine) or had infectious colitis (based on positive stool culture). Patients with any infections, hematological or neoplastic disorders, chronic renal failure, chronic liver or heart diseases or autoimmune diseases were excluded. Another 40 group-matched healthy individuals were recruited for this study and served as a control group. The study protocol was approved by the Research, Ethical Committee of Ain Shams University. All participants did sign an informed written consent.

### Study design

All participants were subjected to detailed history and clinical examination with special prominence on abdominal pain, significant weight loss, rectal bleeding, diarrhea, constipation, nausea, tenesmus, abdominal distension, passage of mucus, vomiting, low-grade fever, and positive family history of inflammatory bowel diseases. Complete blood count with differential leukocyte ratios (NLR and LMR), CRP, ESR, total serum protein, serum albumin and stool analysis were done as routine laboratory investigations. Stool culture and sensitivity to exclude the presence of infection were done whenever needed. Radiological investigations including abdominal ultrasound to exclude the presence of associated diseases or complications were performed.

### Colonoscopy and Mayo score

Total colonoscopy with intubation of terminal ileum was performed and multiple biopsies were taken. The disease was divided according to the site and extent of the colonic involvement as the following according to the Montreal classification: ulcerative proctitis, proctosigmoiditis, left side colitis, extensive colitis (> splenic flexure) and pancolitis. The endoscopic assessment of ulcerative colitis was categorized according to the endoscopic pictures as the following: inactive disease (normal mucosa), mild (erythema, decreased vascular pattern, mild friability), moderate (marked erythema, erosions, marked friability) and severe (spontaneous bleeding, ulcerations, pseudo-polyps). The disease activity of UC was evaluated using Mayo UC score and the disease was considered active if the score was > 2 and in remission if the score was = 0–1 [20].

### White blood cells (WBC) with differential count (NLR and LMR)

Complete blood picture with WBC differential was analyzed using a Coulter counter (T660; Beckman Coulter, Brea, CA). Each type of leukocyte was expressed as a percent of the total number of WBCs. The percent value was multiplied by the total leukocytic count to calculate the absolute value. The NLR was calculated from the differential count by dividing the absolute neutrophil count by the absolute lymphocyte count. The LMR was calculated from the differential count by dividing the absolute lymphocyte count by the absolute monocyte count.

### Statistical analysis

Data management and analysis were performed using IBM Statistical Package of Social Sciences (SPSS) version 23, USA. Descriptive data were generated for all variables. Qualitative data were expressed as (numbers and percentages) and compared using Chi-square test. Quantitative data were expressed as mean ± standard deviation for parametric and median with inter-quartile range (IQR) for non-parametric. Comparison between more than two groups with quantitative data of parametric distribution was done by using One Way ANOVA test followed by post hoc analysis using LSD test. Comparison between more than two groups with quantitative data of non-parametric distribution was done by using Kruskal–Wallis test followed by post hoc analysis using Mann–Whitney test. Comparison between two groups with quantitative data of parametric distribution was done by Independent t-test. Spearman’s correlation was used to analyze the correlation between quantitative parameters. A multivariate logistic regression analysis model was applied to take the activity as the dichotomous outcome to assess predictors of activity with its odds ratio and 95% confidence interval. Receiver operating characteristic curve (ROC) was used to assess the best cut off value with its sensitivity, specificity, positive predictive value, negative predictive value and area under the curve (AUC) for the predictors of activity. The confidence interval was set to 95% and the margin of error accepted was set to 5%. So, the p values < 0.05 was considered statistically significant [21].

## Results

### Demographic data and characteristics of the study groups

We enrolled 80 patients with UC (40 with active UC vs 40 with inactive UC) and 40 healthy individuals. Demographic of UC subjects and controls are shown in Table 1. Age and gender were comparable between the study groups and no significant findings could be detected. The distribution of the disease in the colon and endoscopic pattern were displayed in Table 1.

**Table 1:** Demographic data, distribution of the disease in the colon and endoscopic pattern in UC patients

	UC (80) No. %	Controls (40) No. %	p value
Gender			
Males	34 (42.5%)	28 (70%)	0.058^a^
Females	46 (57.5%)	12 (30%)	
Age (mean ± SD)	34 ± 9.449	33.9 ± 8.723	0.969^b^

Ulcerative colitis cases (n = 80)		No.	%
Disease involvement	Proctosigmoid	28	35
	Left side colon	34	42.5
	Extensive	16	20.0
	Pancolitis	2	2.5
Endoscopic picture	Normal/inactive	40	50.0
	Mild	6	7.5
	Moderate	24	30.0
	Severe	10	12.5

*UC* ulcerative colitis, *SD* standard deviation

^a^Chi-square test

^b^Independent t-test

### Laboratory parameters of the study groups

The results, as shown in Table 2 indicated significantly higher WBC, absolute neutrophilic count, absolute monocytic count, NLRs, CRP, and ESR in active UC group compared to both inactive UC patients and controls. While, it indicated significant decrease of absolute lymphocytic count and LMRs in active UC compared to inactive UC patients and controls. The mean NLRs in the three study groups were (active UC: 2.63 ± 0.43 vs inactive UC: 1.64 ± 0.25 vs controls: 1.44 ± 0.19; p < 0.0001). The mean LMRs in the three study groups were (active UC: 2.25 ± 0.51 vs inactive UC: 3.58 ± 0.76 vs controls: 3.64 ± 0.49; p < 0.0001).

**Table 2:** Laboratory parameters of the study groups (control, inactive and active ulcerative colitis)

	Control	Inactive	Active	Test value	p-value	Post hoc analysis		
	No. = 40	No. = 40	No. = 40			P1	P2	P3
Hb (g/dL)								
Mean ± SD	14.09 ± 1.01	12.79 ± 1.19	12.44 ± 1.71	17.299^a^	0.000	0.000	0.000	0.232
Range	12.7–16.2	10.1–15	8.6–14.1					
Platelet								
Mean ± SD	314.90 ± 68.50	303.10 ± 68.30	304.55 ± 77.42	0.332^a^	0.718	0.456	0.513	0.927
Range	187–413	194–413	178–411					
ESR (mm/h)								
Median (IQR)	10 (6–12)	18.5 (16.5–33)	28.5 (19–46)	63.411^b^	0.000	0.000	0.000	0.037
Range	2–19	10–70	12–80					
CRP (mg/dL)								
Median (IQR)	0 (0–1)	2 (1–4.5)	20.5 (16–32.5)	90.859^b^	0.000	0.000	0.000	0.000
Range	0–6	0–11	6–65					
WBC								
Mean ± SD	5.94 ± 1.41	6.49 ± 1.41	7.54 ± 2.06	9.820^a^	0.000	0.140	0.000	0.005
Range	3.9–8.59	4.55–9.49	4.21–10.29					
Absolute neutrophil								
Mean ± SD	3.26 ± 0.87	3.62 ± 0.92	4.84 ± 1.46	22.566^a^	0.000	0.147	0.000	0.000
Range	2.01–5.19	2.4–5.72	2.6–7					
Absolute lymphocytic								
Mean ± SD	2.27 ± 0.56	2.24 ± 0.53	1.87 ± 0.59	6.491^a^	0.002	0.751	0.001	0.004
Range	1.5–3	1.5–3	1–3					
Absolute monocytic								
Mean ± SD	0.40 ± 0.11	0.63 ± 0.07	0.82 ± 0.15	145.993^a^	0.000	0.000	0.000	0.000
Range	0.25–0.62	0.5–0.77	0.5–1					
NLR								
Mean ± SD	1.44 ± 0.19	1.64 ± 0.25	2.63 ± 0.43	179.622^a^	0.000	0.004	0.000	0.000
Range	1.2–1.83	1.28–2.12	1.81–3.21					
LMR								
Mean ± SD	3.64 ± 0.49	3.58 ± 0.76	2.25 ± 0.51	69.838^a^	0.000	0.625	0.000	0.000
Range	2.6–4.82	2.27–4.98	1.35–.03					

P1: control vs inactive; P2: control vs active; P3: active vs inactive

*ESR* erythrocyte sedimentation rate, *CRP* C-reactive protein, *WBC* Wight blood cells; *NLR* neutrophil lymphocyte ratio, *LMR* lymphocyte monocyte ratio

^a^One Way ANOVA followed by post hoc using LSD

^b^Kruskall–Wallis test followed by post hoc using Mann–Whitney

^c^Independent t-test

### Logistic regression analysis for predictors of active disease

As shown in Table 3, multivariate logistic regression analysis was used to delineate the associations of the WBC, NLR, LMR, CRP, and ESR with active UC. After adjusting for other inflammatory markers (WBC, CRP, and ESR), the odds ratio of the NLR was 35.23 (95% confidence interval, (7.54–165.244) and the odds ratio of the LMR was 0.015 (95% confidence interval, (0.002–0.104). The multivariable analysis revealed that NLR, could be a parameter capable of discriminating active from inactive UC.

**Table 3:** Multivariate logistic regression analysis for the relation between NLR and LMR with disease activity of UC as adjusted for possible confounding factors (no. = 80)

	B	S.E.	Wald	p-value	Odds ratio (OR)	95% CI for OR	
						Lower	Upper
ESR	0.025	0.013	3.805	0.051	1.025	1.000	1.051
CRP	0.621	0.174	12.798	0.000	1.861	1.324	2.616
WBC	0.343	0.135	6.423	0.011	1.409	1.081	1.836
NLR	8.210	2.176	14.234	0.000	36.773	15.679	61.290
LMR	− 4.176	0.977	18.265	0.000	0.015	0.002	0.104

*CI*  confidence interval, *ESR* erythrocyte sedimentation rate, *CRP* C-reactive protein; *WBC* white blood cells, *NLR* neutrophil–lymphocyte ratio, *LMR* lymphocyte-monocyte ratio

### Receiver operating characteristic curve (ROC) for predictors of active disease

ROC analysis detected that the cut-off value of the NLR for active UC was **> **1.91, with sensitivity and specificity of 90% and 90% respectively. The cut-off value of the LMR for active UC was ≤ 2.88, with sensitivity and specificity of 90% and 90% respectively. The cut-off value of CRP for determining active disease was > 9 with a sensitivity of 95% and a specificity of 95%. The cut-off value of ESR for determining active disease was > 19 with sensitivity and specificity of 75% and 55% respectively (Table 4).

**Table 4:** Diagnostic performance of the studied inflammatory markers in diagnosis of active ulcerative colitis (no. = 80)

	Cut off point	AUC	Sensitivity (%)	Specificity (%)	PPV	NPV
ESR	> 19	0.635	75	55	62.5	68.7
CRP	> 9	0.986	95	95	95%	95%
WBC	> 6.67	0.660	75	70	71.4%	73.7%
NLR	> 1.91	0.971	90	90	90%	90%
LMR	≤ 2.88	0.949	90	90	90%	90%

*AUC* area under curve, *PPV* positive predictive value, *NPV* negative predictive value

### Correlation of NLR and LMR with other inflammatory markers

Spearman’s correlation analysis revealed significant positive correlations between NLR and WBC (r = 0.324, p = 0.012), ESR (r = 556, p = 0.00) and CRP (r = 789, p = 0.00) in the sum of all UC patients. Closer inspection of Table 5 show a positive correlation between NLR with ESR (r = 0.597, p = 0.005) and CRP (r = 0.490, p = 0.082) in patients with active UC; however, no correlation was found between the NLR CRP, ESR or WBC count in patients with inactive disease. As regard LMR, Spearman’s correlation analysis reported significant negative correlations between LMR with ESR (r = − 0.525, p = 0.00) and CRP (r = − 0.682, p = 0.00) in sum of all UC patients. A negative significant correlation between LMR with ESR (r = − 0.489, p = 0.029) and CRP (r = − 0.475, p = 0.034) was found in patients with active UC; however, no correlation was found between the LMR and CRP, ESR or WBC count in patients with inactive disease (Table 5).

**Table 5:** Spearman correlation coefficients between both NLR or LMR and the other studied inflammatory markers in patients with UC

	WBC		ESR (mm/h)		CRP (mg/dL)	
	R	p-value	R	p-value	r	p-value
	*Neutrophil/lymphocyte ratio*					
All patients (no. = 80)	0.324*	0.012	0.556**	0.000	0.789**	0.000
Active (no. = 40)	0.184	0.437	0.597**	0.005	0.490*	0.028
Inactive (no. = 40)	0.088	0.712	− 0.139	0.558	0.146	0.538
	*Lymphocyte/monocyte ratio*					
All patients (no. = 80)	− 0.038	0.774	− 0.525**	0.000	− 0.682**	0.000
Active (no. = 40)	− 0.018	0.804	− 0.489*	0.029	− 0.475*	0.034
Inactive (no. = 40)	− 0.276	0.239	− 0.21	0.373	− 0.214	0.366

Spearman correlation coefficients

* Significant, ** Highly significant

### Relation of disease severity and extent in active UC group with N/L and L/M ratios

Interestingly, NLR and LMR were not only found to be good predictors of disease activity in UC patients, but also, NLR was significantly higher in more extensive and more severe ulcerative colitis while LMR was found to be significantly lower in more extensive ulcerative colitis as set out in Table 6.

**Table 6:** NLR and LMR in relation to the endoscopic disease activity and the extent of disease

	Mild	Moderate	Severe	One Way ANOVA	p-value
	No. = 12	No. = 25	No. = 3	F	
NLR					
Mean ± SD	2.18 ± 0.22	2.80 ± 0.33	3.11 ± 0.08	22.083	0.000
Range	1.81–2.47	1.9–3.21	3.06–3.2		
LMR					
Mean ± SD	2.44 ± 0.47	2.18 ± 0.52	2.16 ± 0.22	1.198	0.313
Range	1.63–3.03	1.35–3	1.91–2.29		

	Proctosigmoid-left side colon	Extensive-pancoloitis	Independent t-test	p-value
	No. = 62	No. = 18	T	
NLR				
Mean ± SD	1.95 ± 0.51	2.78 ± 0.46	6.178	0.000
Range	1.28–3.2	1.61–3.21		
LMR				
Mean ± SD	3.08 ± 0.91	2.35 ± 0.73	3.128	0.002
Range	1.35–4.98	1.67–3.98		

## Discussion

Assessment of ulcerative colitis depends on clinical manifestations together with radiological investigations, endoscopic and histopathological examination. Endoscopy still is essential for diagnosis of ulcerative colitis [22]. Unfortunately it may not always be applicable due to possible complications in active ulcerative colitis, or its unavailability. The initial objective is to search for other alternatives to evaluate these patients and achieve the main aim of treatment which is endoscopic and clinical remission [23].

Hence, simple non-invasive biomarkers are needed to avoid the disadvantageous complications of invasive diagnostic procedures. Previously studied biomarkers for predicting activity in UC, are either serum markers (e.g. CRP and ESR or serological and antibody markers) or fecal markers (e.g. calprotectin and lactoferrin). Serum markers have low sensitivity and specificity and so are inefficient in reflecting disease activity [24]. On the other side, fecal marker calprotectin is the best clinically available biomarker for UC activity with a sensitivity of 93% and specificity of 96% [25]. However, its use is limited in routine practice due to high cost, prolonged time of sample processing, and inability to collect stool samples [26].

Various studies have assessed the efficacy of circulating blood leukocyte subtypes as biomarkers of inflammatory disorders, including IBD [5, 8–11, 27, 28]. They established that both of absolute neutrophil and lymphocyte counts and their ratios are significantly correlated with the activity in UC. In addition, complete blood counts with differential are almost ordered at clinic visit of IBD patients and hence routinely available. Thus the rationale behind the current study was to elucidate the role of differential leucocyte ratio (NLR and LMR) as a simple, and available predictors of disease activity in patients with ulcerative colitis and also to determine their possible association with the pattern of colonic involvement and endoscopic severity.

We detected findings that confirm and extend multiple previous studies. During routine follow up, the most commonly utilized serum markers to assess active disease are ESR and CRP. In terms of differentiating disease activity, our results emphasized that serum levels of CRP and ESR were significantly higher in patients with active UC compared with non-active UC and controls (p < 0.0001). By using, ROC curve analysis, we found that the cut off value for CRP and ESR was 9 and 19 respectively, with sensitivity and specificity of 95% and 95% and 75% and 55% respectively. Our results are consistent with those of Solem et al. [5] who stated that in UC patients, elevated CRP was significantly associated with severe clinical activity, elevation in ESR, and active disease, but not with histologic inflammation.

The current study demonstrated significant elevation of both absolute neutrophilic count and NLR in patients with active UC compared with inactive UC patients and controls while the absolute lymphocytic count was significantly lower. Moreover, in the multivariate logistic regression analysis, NLR together with CRP were found to be an independent markers that were capable of differentiating active UC from inactive UC. These results match those observed in earlier studies.

Neutrophil is one of the most important cells infiltrating the intestinal mucosa during inflammation. Excess infiltration occurs as result of induced recruitment of neutrophils and defect of apoptosis in UC. Hence, it has a significant role in the development of mucosal inflammation seen in UC. Neutrophils infiltration in the mucosa helps to remove cellular debris and clear bacteria that may contaminate intestinal wounds. Besides, these recruited neutrophils release defensins and cathelicidin to stimulate the migration and proliferation of epithelial cells, and enhance the production of protective mucins [29]. Surprisingly, neutrophils also can facilitate intestinal repair by inducing proteins and lipid mediators [30]. This explains the increased absolute neutrophilic count in active UC.

Prior studies in both Crohn’s disease and ulcerative colitis have detected the presence of lymphocytes malfunction (reduction in lymphocyte responsiveness to the mitogen phytohemagglutinin) at both the peripheral and mucosal level [27, 31, 32]. Hence, NLR can represent two arms of the immune system, neutrophils that represent the existence of inflammation and lymphocytes that signify the regulatory pathway [33, 34]. Thus, the NLR can be an indicator of the presence of ongoing inflammation, and this can clarify increased NLR in active UC patients.

In accordance with the present results, previous studies have demonstrated significantly elevated NLR in patients with active UC [18, 24, 27]. It has previously been observed that 2.16–3.1 is the optimal NLR cutoff value for active UC [17, 18, 24, 27]. Torun and colleagues conducted a study, including 196 UC patients not on treatment (119 are active and 77 are inactive) compared to 59 group-matched healthy individuals. They detected raised NLR values in active UC compared to inactive UC patients and controls. By the ROC analysis, the cut-off value of 2.16 with a sensitivity of 81.8% and a specificity of 80.5% indicated active UC [27]. These data obtained by Torun et al. supported ours.

Likewise, Akpinar and colleagues investigated the sensitivity of NLR to predict endoscopic disease severity in 104 patients with active UC, 104 patients in remission, and 105 healthy individuals. The mean NLR were significantly higher in the active group compared to the other study groups (p < 0.001). They detected that NLR can identify activity and were associated with mucosal injury [28]. This goes in line with our study however our patients were newly diagnosed and this may affect the different cut off value of NLR. Also Rosenberg et al. [35] showed that leukocytes and CRP have an ability to predict the presence endoscopically active UC.

In contrast, 71 patients with UC and 140 healthy individuals (control group) were enrolled in a study by Demir et al. [18], in which the NLR values of the active UC group were elevated compared with those of the patients with inactive UC and the controls (p = 0.005). In their results, the ROC analysis revealed 2.39 as the optimum NLR cut-off value for active UC with a sensitivity of 48.6 and specificity of 77.5%. Hence, they concluded that only CRP was able to significantly differentiate active from inactive UC due to the low sensitivity and specificity rates of NLR in determining active UC compared to that of the CRP (63% and 57% respectively).

The findings of the current study seem to be consistent with those of Cherfane and colleagues who conducted a study, including 110 UC patients, 75 patients infected with C. Difficile, and 75 non-IBD individuals. The authors concluded that the NLR was effective to distinguish active UC from non-IBD controls, but not from quiescent UC [19].

The current study found significant elevation of absolute monocytic count and decrease of LMR in active UC patients compared with inactive UC patients and controls. The LMR cut off value for determining the UC disease activity was ≤ 2.88 with sensitivity and specificity of 90% and 90%. Also, after adjusting for the other inflammatory markers (ESR, and CRP), the LMR was found also to be an independent marker for discrimination of disease activity.

Monocytes can differentiate into macrophages and dendritic cells in the tissues. During inflammation, pro-inflammatory cytokines and chemokines, can generate monocytes in the bone marrow and recruit them to the site of inflammation. There, they differentiate into tissue resident macrophages and dendritic cells. Persistent activation of monocytes and defective innate immune responses are therefore involved in the development of IBD [29] which can explain the presence of absolute monocytosis in active UC. Thus, monocyte counts are expected to be elevated during active inflammation [36]. These results accord with Cherfane et al. [19] who concluded that monocytosis and a low LMR can identify patients with active UC, however, in their study, NLR values were not significantly different between the two groups.

Interestingly, in the current study NLR was not only found to be an independent marker of disease activity, but also it can predict the endoscopic severity and extent of the disease. There was significant increase of NLR in patients with extensive and pancolitis and high NLR was positively correlated with endoscopic severity (p = 0.000). While, LMR was only significantly lower in patients with extensive and pancolitis (p = 0.002). This outcome is contrary to that of Celikbilek et al. [37] who conducted a study on 26 UC patients and 28 healthy individuals. They detected that, in spite of the significantly increased NLR in patients with active UC patients in comparison to inactive UC and controls (p < 0.001), non-significant difference in inflammatory parameters with disease extension, and activity was found. The optimal NLR cut-off value for active UC was 2.47. Again, our results contradict Cherfane et al. [19] who stated that NLR values were not significantly different between patients with active and those with quiescent colonoscopy.

In conclusion, NLR and LMR had higher sensitivity and specificity than total WBCs and ESR with the benefit of being readily available with an affordable price. They are more useful when utilized together with serum laboratory inflammatory indices (CRP). NLR and LMR were independent indicators of active ulcerative colitis. NLR can also detect the degree and extent of endoscopic involvement. NLR and LMR could be used for assessment of endoscopically active UC activity to reduce the need for invasive endoscopies. NLR and LMR may guide in assessment of UC activity and mucosal injury, when colonoscopy is not available. We do need prospective multi center studies with large cohort of patients with long term follow up with studying various treatment effects on NLR and LMR ratios in UC patients. Besides, it would be prudent to analyze the efficacy of these biomarkers in Crohn’s disease and their role as possible phenotypic markers.
